# Fungal nanoscale metal carbonates and production of electrochemical materials

**DOI:** 10.1111/1751-7915.12765

**Published:** 2017-07-17

**Authors:** Qianwei Li, Geoffrey Michael Gadd

**Affiliations:** ^1^ State Key Laboratory of Heavy Oil Processing Beijing Key Laboratory of Oil and Gas Pollution Control China University of Petroleum 18 Fuxue Road Changping District Beijing 102249 China; ^2^ Geomicrobiology Group School of Life Sciences University of Dundee Dundee DD1 5EH UK

## Abstract

Fungal biomineralization of carbonates results in metal removal from solution or immobilization within a solid matrix. Such a system provides a promising method for removal of toxic or valuable metals from solution, such as Co, Ni, and La, with some carbonates being of nanoscale dimensions. A fungal Mn carbonate biomineralization process can be applied for the synthesis of novel electrochemical materials.

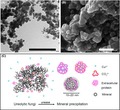

## Carbonate biomineralization

The term biomineralization refers to the collective processes by which organisms form minerals (Gadd, 2010). Biomineralization can be categorized into biologically‐induced mineralization (BIM) and biologically‐controlled mineralization (BCM). BIM occurs when an organism modifies its local microenvironment to create conditions for mineral precipitation, while in BCM complex cellular control mechanisms exist such as in the formation of silicaceous tests in diatoms (Gadd, 2010; Gadd and Raven, 2010; Rhee *et al*., 2012; Kumari *et al*., 2016). Most microbial biomineralization examples refer to biologically induced mineralization. Biomineralization of carbonates has received wide attention. Carbonate minerals, especially the rock‐forming minerals calcite (CaCO_3_) and dolomite (CaMg(CO_3_)_2_), occur in abundance on the Earth's surface as limestones (Burford *et al*., 2006; Ehrlich and Newman, 2008; Lippmann, 2012). Modern mineralogical methods have revealed that a significant proportion of such carbonate minerals at the Earth's surface is of biogenic origin, and many microbial species, including cyanobacteria, bacteria, microalgae and fungi, can deposit calcium carbonate extracellularly (Goudie, 1996; Verrecchia, 2000; Burford *et al*., 2006; Barua *et al*., 2012; Achal *et al*., 2015; Kumari *et al*., 2016). Carbonates of calcium and other metals are also significant substances used in a wide variety of industrial and agricultural applications. The process of microbial carbonate biomineralization has been investigated as a promising bioremediation strategy for toxic metal immobilization in soil (Kumari *et al*., 2016; Zhu *et al*., 2016a) as well as soil stabilization and the development of biocements and biogrouts for construction purposes (Achal *et al*., 2015; Li *et al*., 2015a,b). It is now known that some carbonate biominerals may be deposited in nanoscale dimensions (Li *et al*., 2014, 2016), providing further significant physical, chemical and biological properties of applied significance (Hochella *et al*., 2008). This article will describe the potential applications of fungal‐mediated metal carbonate bioprecipitation including the development of new electrochemical materials.

## Carbonate biomineralization of toxic or valuable metals

**Figure 1:**
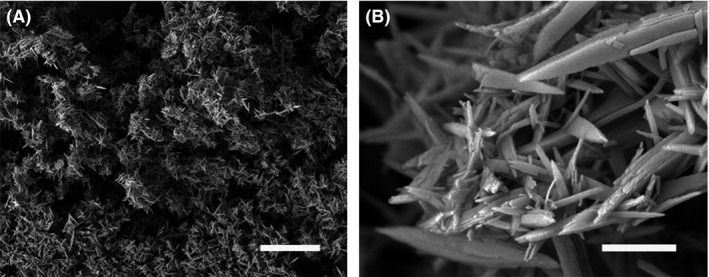
Scanning electron microscopy images of lanthanum carbonate precipitated on addition of LaCl_3_ to a culture supernatant derived from growth of *Neurospora crassa* in urea‐supplemented medium. Scale bars: (A) = 20 μm, (B) = 1 μm. Typical images are shown from many similar examples (Li, Q. and Gadd, G.M., unpublished).

Fungal biomineralization of carbonates results in metal removal from solution or immobilization within a solid matrix providing a method for detoxification as well as recovery (Table 1). Biologically‐induced mineralization (BIM) involving urea hydrolysis by urease‐positive microorganisms, which leads to metal carbonate precipitation, has been found to be effective in immobilizing several potentially toxic metals, for example Cd, Ni, Pb, Sr, and the metalloid As (Achal, 2012; Achal *et al*., 2012; Li *et al*., 2014, 2015a,b; Zhu *et al*., 2016a). Urease‐positive fungi, such as *N. crassa*, have the ability to precipitate metal carbonates in the media and around the biomass when incubated in urea‐amended media while culture supernatants also provide a biomass‐free carbonate bioprecipitation system (Li *et al*., 2014, 2015a,b). In a novel application of calcium carbonate biomineralization, Li *et al*. (2014) demonstrated that supplied cadmium could be precipitated as pure otavite (CdCO_3_) by culture supernatants derived from growth of *Neurospora crassa* in urea‐supplemented medium. A new lead hydroxycarbonate was precipitated by *Paecilomyces javanicus* grown in medium containing metallic lead. Other secondary lead minerals precipitated included plumbonacrite (Pb_10_(CO_3_)_6_O(OH)_6_) and hydrocerussite (Pb_3_(CO_3_)_2_(OH)_2_) (Rhee *et al*., 2012). The advantage of using ureolytic microorganisms for toxic metal immobilization is their ability to efficiently immobilize metals in carbonate minerals by precipitation or co‐precipitation regardless of the metal valence state and toxicity, and the redox potential (Kumari *et al*., 2016). It has been suggested that such a system may also provide a promising method for removal of toxic or valuable metals from solution, such as Co, Ni and La. On addition of LaCl_3_ to carbonate‐laden fungal culture supernatants, fusiform‐shaped lanthanum carbonate was precipitated with approximate sizes ranging from 1 to 5 μm (Fig. 1). This is the first report of lanthanum biorecovery using geoactive fungal growth supernatants. Lanthanum, as one of the rare earth elements (REE), plays an important role in advanced new materials, such as superalloys, catalysts, specialized ceramics and organic synthesis (Kanazawa and Kamitani, 2006; Das and Das, 2013). Conventional chemical methods for La extraction are based on hydrometallurgy combined with a pyrometallurgical process which are energy intensive and produce significant amounts of chemical sludge at the same time (Wang *et al*., 2011; Das and Das, 2013). Various biosorbents including macroalgae (Diniz and Volesky, 2005) and bacteria (Kazy *et al*., 2006) have also been applied for lanthanum although, despite years of research, the credibility of metal biosorption as a commercially viable technique is very limited (Gadd, 2009).

**Table 1:** Biorecovery of toxic or valuable metals by fungal carbonate biomineralization

Metal	Fungal species	Precipitated metal carbonate	References
Ba	*Verticillium* sp.	BaCO_3_	Rautaray *et al*. (2004)
Cd	*Fusarium oxysporum, Neurospora crassa, Myrothecium gramineum, Pestalotiopsis* sp.	CdCO_3_	Sanyal *et al*. (2005); Li *et al*. (2014)
Co	*N. crassa, M. gramineum, Pestalotiopsis* sp.,	CoCO_3_∙xH_2_O	Li, Q. and Gadd, G.M., unpublished
Cu	*N. crassa, M. gramineum, Pestalotiopsis* sp.,	Cu_2_(OH)_2_CO_3_, Cu_3_(OH)_2_(CO_3_)_2_	Li, Q. and Gadd, G.M., unpublished
La	*N. crassa, M. gramineum, Pestalotiopsis* sp.	La_2_(CO_3_)_3_·8H_2_O	Li, Q. and Gadd, G.M., unpublished
Ni	*N. crassa, M. gramineum, Pestalotiopsis* sp.	NiCO_3_∙xH_2_O	Li, Q. and Gadd, G.M., unpublished
Pb	*F. oxysporum, Paecilomyces javanicus*	PbCO_3_, Pb_3_(CO_3_)_2_(OH)_2_, Pb_10_(CO_3_)_6_O(OH)_6_), lead hydroxycarbonate^a^	Sanyal *et al*. (2005); Rhee *et al*. (2015)
Sr	*F. oxysporum, N. crassa, M. gramineum, Pestalotiopsis* sp.	(Ca_x_Sr_1‐x_)CO_3_), Sr(Sr, Ca)(CO_3_)_2_, SrCO_3_	Li and Gadd, unpublished
Zn	*N. crassa, M. gramineum, Pestalotiopsis* sp.	(ZnCO_3_)_2_·(Zn(OH)_2_)_3_	Li and Gadd, unpublished

^a^Precise formula not identified.

**Figure 2:**
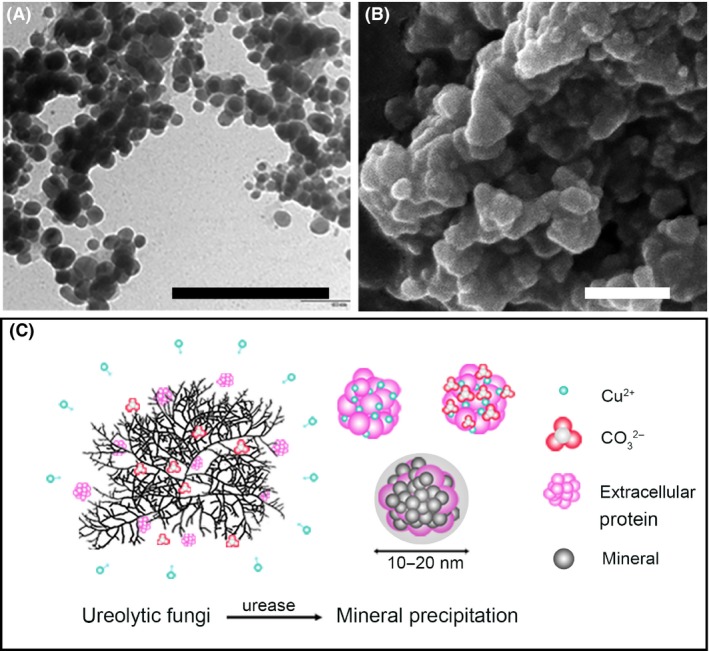
Fungal biomineralization of copper carbonate. A. Transmission electron microscopy image of copper carbonate. B. Scanning electron microscopy of cobalt carbonate both precipitated by addition of the metal chlorides to a culture supernatant derived from growth of *Neurospora crassa* in urea‐supplemented medium. Scale bars = 200 nm. Typical images are shown from many similar examples. C. Model of copper carbonate bioprecipitation in the nanoscale (Li, Q. and Gadd, G.M., unpublished).

Compared to the simpler bacterial cell form, the fungal filamentous growth habit can provide more framework support and stability for the precipitation of carbonates or other biominerals (Kumari *et al*., 2016). Moreover, the physicochemical properties of formed biominerals can also be influenced by biological processes, such as their surface area‐to‐volume ratio, which can show significant differences to bulk minerals (Hochella *et al*., 2008). This is especially true for biominerals that are produced in nanoscale dimensions. The size variation of particles results in differences in surface and near‐surface atomic structure and crystal shape as well as surface topography, which is important in geochemical reactions and kinetics (Hochella *et al*., 2008). Research has demonstrated that many metal‐accumulating or transforming microbes are capable of forming nanoparticles (e.g. Te, Se, CdS, HUO_2_PO_4_) (Macaskie *et al*., 1992; Williams *et al*., 1996; Dickson, 1999; Lloyd *et al*., 1999; Taylor 1999; Klaus‐Joerger *et al*., 2001; Zhu *et al*., 2016b). Their production by microbial systems may allow manipulation of size, morphology, composition and crystallographic orientation, with applications in bioremediation, antimicrobial treatments (e.g. nano‐silver), solar and electrochemical energy, and microelectronics (Dameron *et al*., 1989; Jauho and Buzaneva, 1996; Hayashi *et al*., 1997; Edelstein and Cammaratra, 1998; Klaus‐Joerger *et al*., 2001; Zhu *et al*., 2016b). In a ureolytic fungal‐mediated bioprecipitation system, more than 70% of supplied Co^2+^, Ni^2+^, Cu^2+^ or Zn^2+^ was precipitated in the form of hydrated carbonates and all these minerals showed a nanoscale phase. It appears that fungal metabolites, especially extracellular protein, play an important role in the formation of such nanoscale particles (Fig. 2).

## Carbonate biomineralization for production of electrochemical materials

Increasing consumption and the decline in fossil fuel resources have driven attention to the development of other renewable and sustainable energy sources. Electrical energy storage systems (EESS) such as rechargeable lithium‐ion batteries and electrochemical supercapacitors have shown great promise in this regard (Simon and Gogotsi, 2008; Ji *et al*., 2011; Liu *et al*., 2013; Ding *et al*., 2015). However, performance requirements for these systems are quite critical and Li‐ion batteries have a high specific energy density (energy stored per unit mass) and act as slow and steady energy suppliers for large energy demands. In contrast, supercapacitors possess high specific power (energy transferred per unit mass per unit time) and can charge and discharge quickly for low energy demands. Thus, in the development of electrical energy storage materials, high energy density as well as high power is important (Ding *et al*., 2015). Many efforts have been made to improve the electrochemical performance of supercapacitors or Li‐ion batteries by design of other safe, economic and environment‐friendly electrode materials some of which have a biotic component (Ma *et al*., 2007; Nakayama *et al*., 2007; Sharma *et al*., 2007; Zhu *et al*., 2011; Falco *et al*., 2012; Zhang *et al*., 2012d; Liu *et al*., 2013; Sun *et al*., 2013; Long *et al*., 2015).

Fungal interactions with metals and minerals can alter their physical and chemical state and plays a significant role in environmental element biotransformations and cycling (Kolo *et al*., 2007; Fomina *et al*., 2010; Gadd, 2010; Gadd and Raven, 2010). Fungal hyphae can provide nucleation sites for the precipitation of metals following biosorption, metabolite secretion and/or oxidation or reduction of a metal or metalloid species (Gadd, 2009, 2010). Such processes appear to have potential applications in materials science which hitherto have been rather neglected. Fungal biomass represents an abundant carbon‐neutral renewable resource that can be used for the production of bioenergy and biomaterials, and research has been carried out on the application of biomass (e.g. fungi, bacteria, microalgae) as a carbonaceous electrode material for ESS (Shim *et al*., 2010; Zhu *et al*., 2011; Falco *et al*., 2012). A hydrothermal assisted pyrolysis procedure was applied for the preparation of activated carbon (AC) using crude biomass of an *Auricularia* sp. which exhibited capacitive characteristics (stability, energy density power density, surface capacitance and volumetric capacitance) in supercapacitors. This study provided a facile method for the synthesis of carbonaceous electrode materials and highlighted the potential applications of fungi in materials science (Zhu *et al*., 2011). Similarly, Wang and Liu (2015) used fungal biomass as carbon precursor to prepare hierarchical porous activated carbon (AC), and the fungi‐derived AC electrode showed superior cycling performance in supercapacitors (92% retention after 10 000 cycles). Furthermore, carbonaceous materials with a high porosity obtained from biological cellular structures increases the active carbon surface area which may result in superior electrical properties. They are therefore suggested to be useful electrode materials in micro‐batteries and electrochemical capacitors because of their excellent proton‐ or lithium‐conducting properties (Klaus‐Joerger *et al*., 2001).

Lithium‐ion batteries with high storage capacities and cycling stability are considered to be another promising power source. The performance of a Li‐ion battery is based on the diffusion of Li ions between the anode and the cathode, converting chemical energy to electrical energy which is stored within the battery. For commercial Li‐ion batteries, graphite is the most common anode material due to its low cost and long cycle life. However, some deficiencies of conventional graphite carbon, such as a high sensitivity to the electrolyte and a low charge capacity, can limit the electrochemical performance of Li‐ion batteries. In order to improve the power density and capacity of Li‐ion batteries, various other anode materials have been developed to meet high electrochemical requirements such as carbon nanotubes (CNTs) (Pol and Thackeray, 2011) and manganese oxides (MnO, MnO_2_, Mn_2_O_3_, Mn_3_O_4_), which have excellent electrochemical properties (Xia *et al*., 2013).

It is accepted that the addition of metal oxides to a carbonaceous substrate will increase the electrochemical performance of electrode materials, especially for transition metal oxides (e.g. Co_x_O_y_, V_x_O_y_, Fe_x_O_y_) and those in the nanoscale, with variable oxidation states, are excellent candidates for electrode materials (Poizot *et al*., 2000; Dillon *et al*., 2008; Amade *et al*., 2011; Wu *et al*., 2012; Devaraj *et al*., 2014). Metal carbonates can be very good precursors for preparation of metal oxides. Thus, a fungal Mn biomineralization process based on urease‐mediated manganese carbonate bioprecipitation has been applied for the synthesis of novel electrochemical materials (Li *et al*., 2016). Manganese carbonate encrusted mycelium of *N. crassa* was heat treated (300°C, 4 h) to convert the biomass/precipitated MnCO_3_ to a MnO_x_/C composite material. The electrochemical performance of this biogenic MnO_x_/C was investigated in a hybrid asymmetric supercapacitor as well as in a lithium‐ion battery. The carbonized fungal biomass‐mineral composite (MycMnOx/C) showed a high specific capacitance (> 350 F g^−1^) in a supercapacitor and excellent cycling stability (> 90% capacity was retained after 200 cycles) in a lithium‐ion battery. This was the first demonstration of the synthesis of electrode materials using a fungal biomineralization process and therefore indicates a novel method for the sustainable synthesis of electrochemical materials.

## Future prospects

With the depletion of high‐grade mineral resources and increasing energy costs, adverse environmental effects are becoming more apparent from conventional technologies. Microbial‐based biotechnologies could provide economic alternative methods for the recycling of toxic or valuable metals, and a simplified approach for the synthesis of biomaterials for bioenergy and other applications. Fungal‐mediated metal carbonate precipitation suggests that these organisms can play a role in the environmental fate, bioremediation or biorecovery of metals and radionuclides that form insoluble carbonates and also indicates novel strategies for the preparation of sustainable electrochemical materials and other biomineral products.

## Conflict of interest

None declared.
